# Supplementary material for: Optimal transport analysis reveals trajectories in steady-state systems
Source: PLoS Comput Biol. 2021 Dec 3;17(12):e1009466. doi: 10.1371/journal.pcbi.1009466 (PMC8691649; doi:10.1371/journal.pcbi.1009466)
Supplement: S1 Appendix — Supporting theoretical material and root atlas application details. (PDF) [file pcbi.1009466.s001.pdf]

Supplementary materials

October 30, 2021

1 Supporting theory

1.1 Example illustrating identifiability criterion

Here we present a simple example that illustrates the identifiability issues discussed in Section Introduction. We consider a setting similar to that of Section 2.7, but in 2 dimensions. We impose radial symmetry and work in polar coordinates (r, θ). Let us consider a radial potential, e.g. $\Psi(r)$. We assume that a stationary distribution ρ_{eq} exists. By symmetry, ρ_{eq} has no dependence on θ, i.e. $\rho_{\text{eq}}(r,\theta) = \rho_{\text{eq}}(r)$. A straightforward computation shows that $\nabla\rho_{\text{eq}}(x)$ and $\nabla\Psi(x)$ are multiples of $\hat{\boldsymbol{r}}$. Consider now a vector field that combines the potential gradient with a rotational field

$$\boldsymbol{v}(x) = -\nabla\Psi(x) + r\hat{\boldsymbol{\theta}}.$$

The resulting stationary distribution is still radially symmetric and satisfies (2):

$$0 = -\nabla \cdot (\boldsymbol{v}(x)\rho_{\text{eq}}(x)) + \frac{\sigma^2}{2}\nabla^2\rho_{\text{eq}}(x) + R(x)\rho_{\text{eq}}(x).$$

Focus on $\nabla \cdot (\boldsymbol{v}(x)\rho_{\text{eq}}(x))$ since it is the only term that features $\boldsymbol{v}(x)$. Now, using the product identity for the divergence,

$$\begin{aligned}\nabla \cdot (\boldsymbol{v}(x)\rho_{\text{eq}}(x)) &= -\nabla^2\Psi(x)\rho_{\text{eq}}(x) - \nabla\Psi(x)\cdot\nabla\rho_{\text{eq}}(x) + (\nabla \cdot r\hat{\boldsymbol{\theta}})\rho_{\text{eq}}(x) + r\hat{\boldsymbol{\theta}}\cdot\nabla\rho_{\text{eq}}(x)\\ &= -\nabla^2\Psi(x)\rho_{\text{eq}}(x) - \nabla\Psi(x)\cdot\nabla\rho_{\text{eq}}(x)\end{aligned}$$

Where we have noted that the field $r\hat{\boldsymbol{\theta}}$ is divergence-free and $\nabla\rho_{\text{eq}} \propto \hat{\boldsymbol{r}}$, and so the rotational component removes itself from (2). Thus, both systems with $\boldsymbol{v} = -\nabla\Psi(x)$ and $\boldsymbol{v} = -\nabla\Psi(x) + r\hat{\boldsymbol{\theta}}$ produce the same stationary distribution ρ_{eq}.

More generally as noted by [11], the stationary distribution ρ_{eq} is unchanged upon changing the drift $\boldsymbol{v}$ to $\boldsymbol{v} + \boldsymbol{w}$ whenever $\boldsymbol{w} \cdot \nabla\rho_{\text{eq}} = 0$ everywhere.

1.2 Computation of fate probabilities and conditional MFPT

Fate probabilities As in the main text, we consider a discrete state space $\overline{\mathcal{X}} = \{x_i\}_{i=1}^N$ and take P to be a matrix of transition probabilities. For brevity, let $\overline{\mathcal{X}}_\varnothing$ denote the set of sink states for which we want to compute fate (absorption) probabilities. Then we compute fate probabilities via the fundamental matrix as follows [10, Theorem 28]

- Make all sink states absorbing, i.e. produce a modified transition matrix $\tilde{P}$ from P such that for each sink $x_i \in \overline{\mathcal{X}}_\varnothing$, we have
$$\tilde{P}_{ij} = \mathbf{1}_{i=j}.$$
- We reorder the rows and columns of $\tilde{P}$ to form the matrix
$$\begin{bmatrix} Q & R \\ 0 & I \end{bmatrix}, \tag{1}$$
where Q corresponds to transitions between transient states and R corresponds to transitions from transient states to sink states.

- We next compute the fundamental matrix of the absorbing Markov chain as

$$N = (I - Q)^{-1}. \tag{2}$$

- From the fundamental matrix, we compute $B = NR$. The entry B_{ij} is the absorption probability for sink j for a chain starting from state i, i.e.

$$B_{ij} = \mathbb{P}[X_\infty = x_j | X_0 = x_i].$$

Numerical considerations In practice we have found that the computation of fate probabilities can sometimes suffer from numerical issues that result in the fate probability problem being ill-conditioned. This typically appears to be the case for the quadratic regularisation when the regularisation parameter ε is chosen to be small, resulting in a disconnected graph or degenerate transition laws for outlier cells due to the sparsity of the resulting transport plan. In practice, one can always regularise transition matrices before computing fate probabilities by mixing in a small undirected diffusion component, as is done in [4], i.e.

$$P = (1 - f)P_{\text{statOT}} + fP_{\text{diffusion}},$$

where f is chosen to be small (e.g. $f = 0.05$), and $P_{\text{diffusion}}$ is a matrix of undirected transition probabilities, etc.

$$(P_{\text{diffusion}})_{ij} \propto \exp\left(-\frac{\|x_i - x_j\|^2}{h}\right),$$

and h is a user-specified bandwidth parameter.

Conditional MFPT The conditional mean first passage time (MFPT) from x_i to x_j is defined as

$$\mathbb{E}[t | X_0 = x_i, X_t = x_j].$$

To compute the conditional MFPT, we follow the steps laid out in [11, Theory Supplement, Section 3.3].

Ground truth fate probabilities and ground truth simulation time In simulations, to compute the ground truth fate probability of a state x_i, we simulated 100 trajectories from start to finish, initialised at x_i when $t = 0$. The fate probability of x_i to fate j was taken to be the proportion of simulated trajectories terminating in fate j.

For the ground truth simulation time, we sampled 10^4 trajectories $X_t^{(i)}$ from the underlying process. For each trajectory, we also recorded the simulation time of each state and aggregated these into a reference table. Then, for each sampled state $x_i \in \overline{\mathcal{X}}$, we took the corresponding ground truth simulation time t_i to be the average simulation time of the 250 nearest points in the reference.

1.3 W_2 distances

In Figure 6 we used the 2-Wasserstein (W_2) distance on paths to measure how faithful the inference output was to the ground truth. For probability distributions μ supported on points $\{x_i\}$ and ν supported on $\{y_i\}$, we take a cost matrix C with entries

$$C_{ij} = \frac{1}{2}\|x_i - y_j\|^2.$$

Then, the 2-Wasserstein (W_2) distance is defined as

$$d^2_{W_2}(\mu, \nu) = \inf_{\pi \in \Pi(\mu,\nu)} \sum_{ij} C_{ij}\pi_{ij}. \tag{3}$$

The W_2 distance thus measures how similar μ and ν are, taking into account the underlying Euclidean distances between the support points $\{x_i\}, \{y_i\}$. We refer the reader to the excellent textbook [5] for further details on the W_2 distance and optimal transport.

2 Root atlas application details

2.1 Data preprocessing

The *Arabidopsis thaliana* atlas contains 16 replicates that were preprocessed using the COPILOT pipeline [8]. The replicates were integrated using the Seurat integration pipeline [8, 9]. The resulting atlas was annotated by Shahan et al. with cell lineages and developmental zones using an ensemble of correlation to published expression profiles, Index of Cell Identity scoring, and expression of known marker genes [8, 2]. An ensemble pseudotime was also calculated from RNA velocity using scVelo and based on transcriptional diversity using CytoTRACE [3, 1].

2.2 Estimating Growth Rates

Growth rates were calculated from imaging data of the *Arabidopsis* meristem by averaging the division time observed for all cells in a lineage and converting the division times to an average daily growth rate [6]. In two cases, the imaging data grouped multiple lineages from our data into a single category. In these cases, we used the average growth rate for all cells in that category for each lineage.

As in many cases external growth rates are not available, we estimated alternate growth rates by scoring cells for a cell cycle signature (see Supplement Table 1). In previous applications where cells were rapidly dividing, cell cycle scores were translated into cell-wise growth rates using a logistic function [7]. While we expect all cells in the meristem to undergo division, due to a longer division process we only expect some cells to be undergoing division in our snapshot. So, we took the proportion of cells that expressed the cell cycle signature from each lineage to create growth rates of the relative proportion of cells we expect to be dividing and applied these growth rates to all meristematic cells by lineage. While there were some discrepancies between the growth rates (see Figure A), running StationaryOT with cell cycle growth rates matched 79% and 78% of cells to the annotation for entropic and quadratic regularisation respectively.

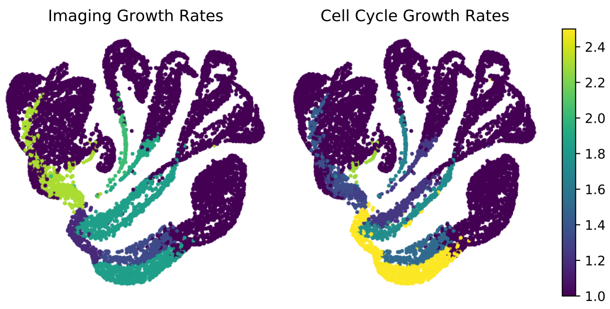

Figure A: Comparison of growth rates by cell between the imaging data (left) and the cell cycle score (right), truncated to 2.5. The largest difference occurred for the quiescent center. In the imaging data, the quiescent center was found to be in the process of division for long periods of time, which explains the low growth rate in the imaging data despite the high cell cycle score. For the other differences, we found the mean cell cycle score to significantly differ by lineage. Using a signature threshold by lineage resulted in growth rates more similar to the imaging growth rates. However, as we may not have tried that approach without the imaging data, we compared to growth rates obtained from a single threshold and still found that StationaryOT's ability to match the annotation only deteriorated by at most 3%.

2.3 Parameter variation

We chose the parameters shown in the main text by performing an initial parameter sweep around a 6-hour time step ($\Delta t = 0.25$) and 5% sinks. The number of sinks was chosen based on the 6-hour time step, the growth rates, and the assumption that the root is in equilibrium so that that number of sinks balanced with the number of cells that would be added by growth. We evaluated the runs based on the percentage of putative fates that matched the annotation. To test the robustness of each method, we tested a grid of parameters taking all combinations of changes of a factor of two and five in each direction.

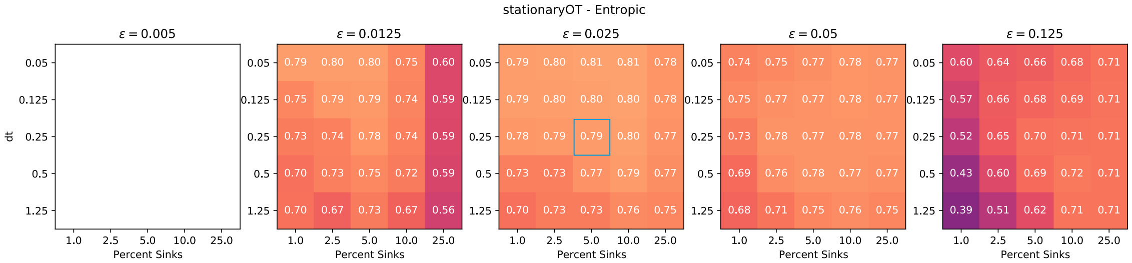

(a)

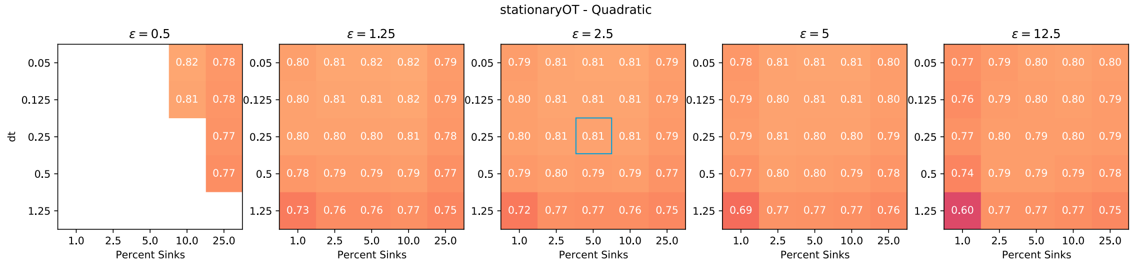

(b)

Figure B: Parameter variation for StationaryOT with (a) entropic and (b) quadratic regularisation. The value for each combination represents the percentage of putative fates matching the annotation. Blank squares indicate runs where fates could not be calculated due to underflow or a singular coupling. The run shown in the main text is indicated in blue.

For StationaryOT with entropic regularisation and small values of ε, we added a small amount of Gaussian noise to the coupling to avoid a few outlier cells remaining stationary. As shown in Figure Ba, the match to the annotation changed no more than 2% when changing a parameter by a factor of two, but performance significantly degraded when multiple factors were off by a factor of two or more. StationaryOT with quadratic regularisation was more robust to parameter changes, differing by no more than 2% for a factor of two change in a single parameter and remaining within 2% for many combinations where multiple parameters were off by a factor of five. When using quadratic regularisation with very small values of ε, the coupling matrix becomes singular and we cannot calculate fate probabilities. When using entropic regularisation for very small values of epsilon, the coupling could not be computed due to underflow. For both of these issues, runs are shown as blank squares in Figure Bb.

Like StationaryOT, PBA has parameters for the number of sinks and time step. However, instead of using a single regularisation parameter, PBA has both diffusivity (D) and number of neighbours in the k-NN graph (k). To limit the number of runs to 256, D and k were fixed while the other three parameters were varied (see Figures Ca, Cb). We found that PBA was highly sensitive to the number of sinks in addition to the total flux. Using our estimate that the root would stay in equilibrium when 5% of cells were set as sinks for a 6-hour time step, around 70% of cells matched the annotation. Reducing the number of sinks to 1% for a 6-hour interval increased the match to 80%. However, in many applications the annotation would not be available to evaluate the runs, and the best combination of parameters could be missed.

CellRank has parameters for the number of neighbours in the k-NN graph, the weight on the transcriptional similarity kernel, and the weight on the softmax norm. Based on a parameter sweep, we chose $k = 10$ for the k-NN graph, 0.5 for the weight on the transcriptional similarity kernel, and a softmax of 7.5 (see Figure Cc). With these parameters, CellRank was unable to automatically detect all terminal and initial macrostates (see S4 Fig). Therefore, macrostates were assigned manually to the same sinks used for PBA and StationaryOT. Sources were defined to be all cells in the stem cell niche and the putative quiescent centre.

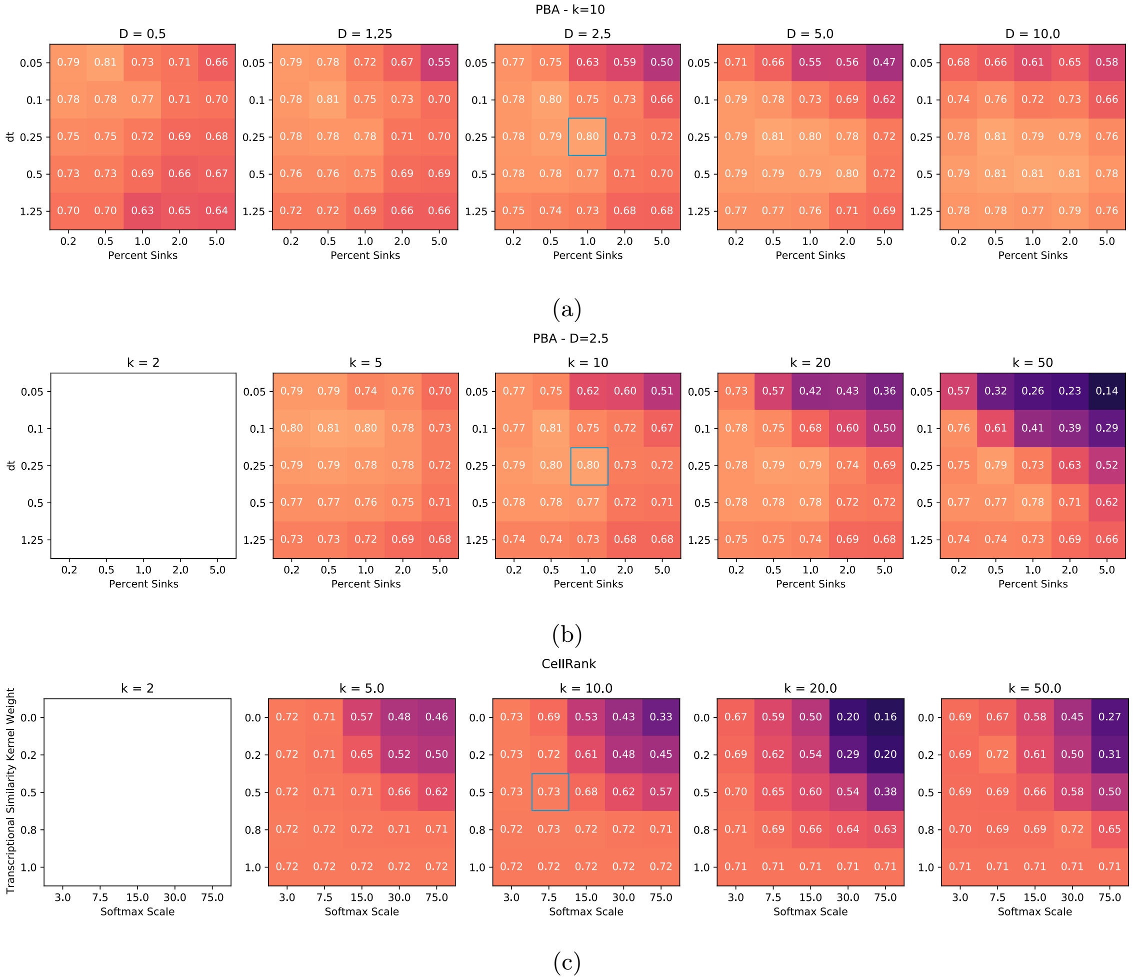

Figure C: Parameter variation for PBA (a-b) and CellRank (c), evaluated by the percentage of cells whose putative fate matched the annotation. For both PBA and CellRank, the graphs became disconnected when only two neighbours were used and thus fate probabilities could not be computed. The run shown in the main text is indicated in blue.

References

[1] Volker Bergen, Marius Lange, Stefan Peidli, F Alexander Wolf, and Fabian J Theis. Generalizing rna velocity to transient cell states through dynamical modeling. *Nature Biotechnology*, pages 1–7, 2020.

[2] Idan Efroni, Pui-Leng Ip, Tal Nawy, Alison Mello, and Kenneth D Birnbaum. Quantification of cell identity from single-cell gene expression profiles. *Genome Biology*, 16(9).

[3] Gunsagar S. Gulati, Shaheen S. Sikandar, Daniel J. Wesche, Anoop Manjunath, Anjan Bharadwaj, Mark J. Berger, Francisco Ilagan, Angera H. Kuo, Robert W. Hsieh, Shang Cai, Maider Zabala, Ferenc A. Scheeren, Neethan A. Lobo, Dalong Qian, Feiqiao B. Yu, Frederick M. Dirbas, Michael F. Clarke, and Aaron M. Newman. Single-cell transcriptional diversity is a hallmark of developmental potential. *Science*, 367(6476):405–411, 2020.

[4] Marius Lange, Volker Bergen, Michal Klein, Manu Setty, Bernhard Reuter, Mostafa Bakhti, Heiko Lickert, Meshal Ansari, Janine Schniering, Herbert B. Schiller, Dana Pe'er, and Fabian J. Theis. Cellrank for directed single-cell fate mapping. *bioRxiv*, 2020.

[5] Gabriel Peyré, Marco Cuturi, et al. Computational optimal transport: With applications to data science. *Foundations and Trends in Machine Learning*, 11(5-6):355–607, 2019.

[6] Rahim Rahni and Kenneth D Birnbaum. Week-long imaging of cell divisions in the arabidopsis root meristem. *Plant Methods*, 15(30), 2019.

[7] Geoffrey Schiebinger, Jian Shu, Marcin Tabaka, Brian Cleary, Vidya Subramanian, Aryeh Solomon, Joshua Gould, Siyan Liu, Stacie Lin, Peter Berube, et al. Optimal-transport analysis of single-cell gene expression identifies developmental trajectories in reprogramming. *Cell*, 176(4):928–943, 2019.

[8] Rachel Shahan, Che-Wei Hsu, Trevor M Nolan, Benjamin J Cole, Isaiah W Taylor, Anna Hendrika Cornelia Vlot, Philip N Benfey, and Uwe Ohler. A single cell arabidopsis root atlas reveals developmental trajectories in wild type and cell identity mutants. *bioRxiv*, 2020.

[9] Tim Stuart, Andrew Butler, Paul Hoffman, Christoph Hafemeister, Efthymia Papalexi, William M 3rd Mauck, Yuhan Hao, Marlon Stoeckius, Peter Smibert, and Rahul Satija. Comprehensive integration of single-cell data. *Cell*, 177(7):1888–1902, 2019.

[10] Anders Tolver. An introduction to markov chains. *Department of Mathematical Sciences, University of Copenhagen*, 2016.

[11] Caleb Weinreb, Samuel Wolock, Betsabeh K Tusi, Merav Socolovsky, and Allon M Klein. Fundamental limits on dynamic inference from single-cell snapshots. *Proceedings of the National Academy of Sciences*, 115(10):E2467–E2476, 2018.
