# Supplementary figures and images for: Optimal transport analysis reveals trajectories in steady-state systems
Source: PLoS Comput Biol. 2021 Dec 3;17(12):e1009466. doi: 10.1371/journal.pcbi.1009466 (PMC8691649; doi:10.1371/journal.pcbi.1009466)

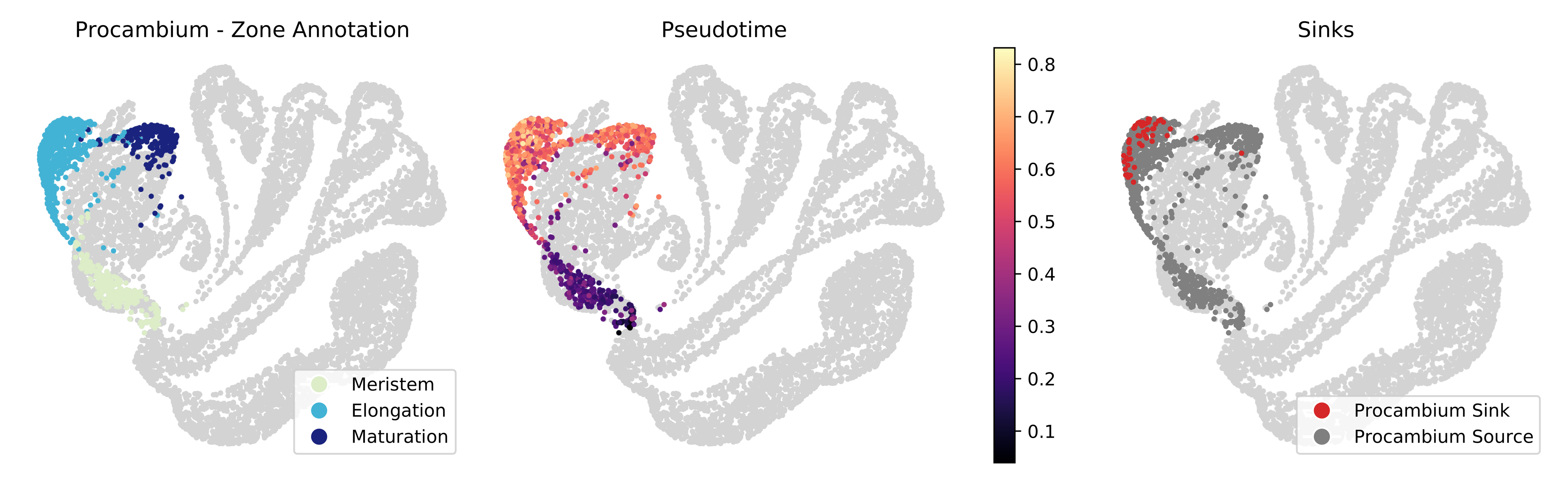

Supplement: S1 Fig — We believe this occurred due to a disagreement between the pseudotime and zone annotations, where procambium cells in the elongation zone were given a higher pseudotime than those in the maturation zone, resulting in cells from the elongation zone incorrectly being set as terminal states. (TIF) [file pcbi.1009466.s003.tif]

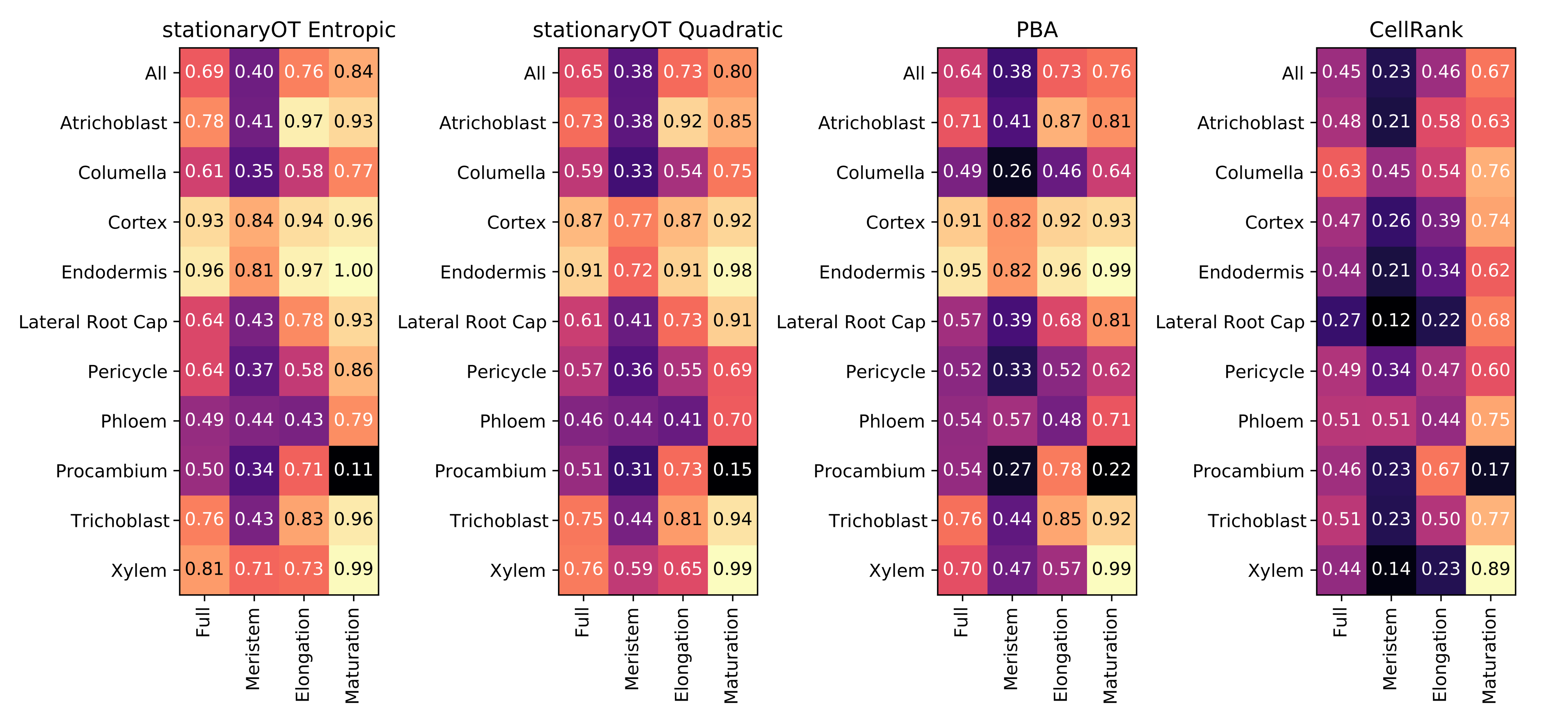

Supplement: S2 Fig — (TIF) [file pcbi.1009466.s004.tif]

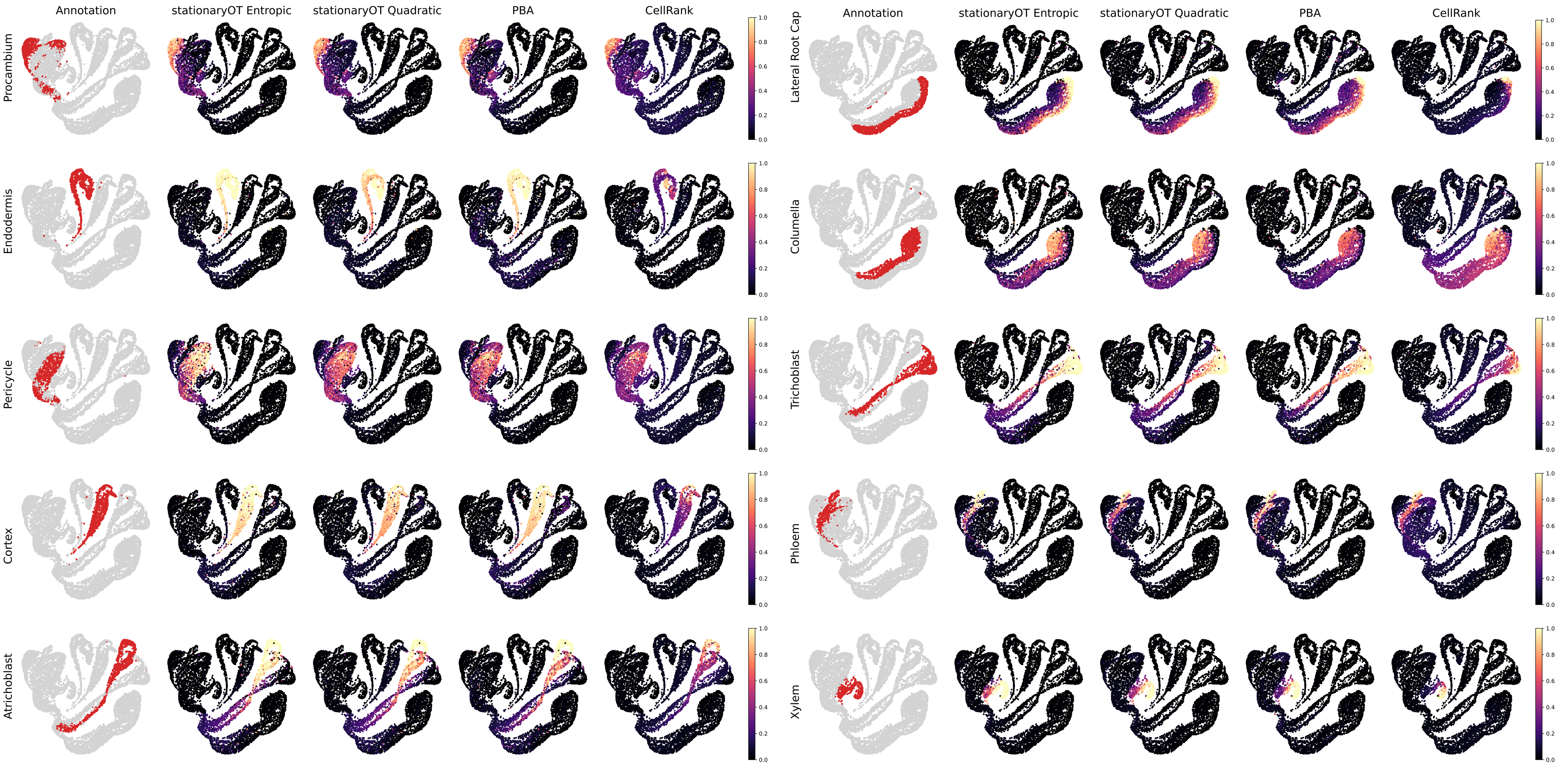

Supplement: S3 Fig — (TIF) [file pcbi.1009466.s005.tif]

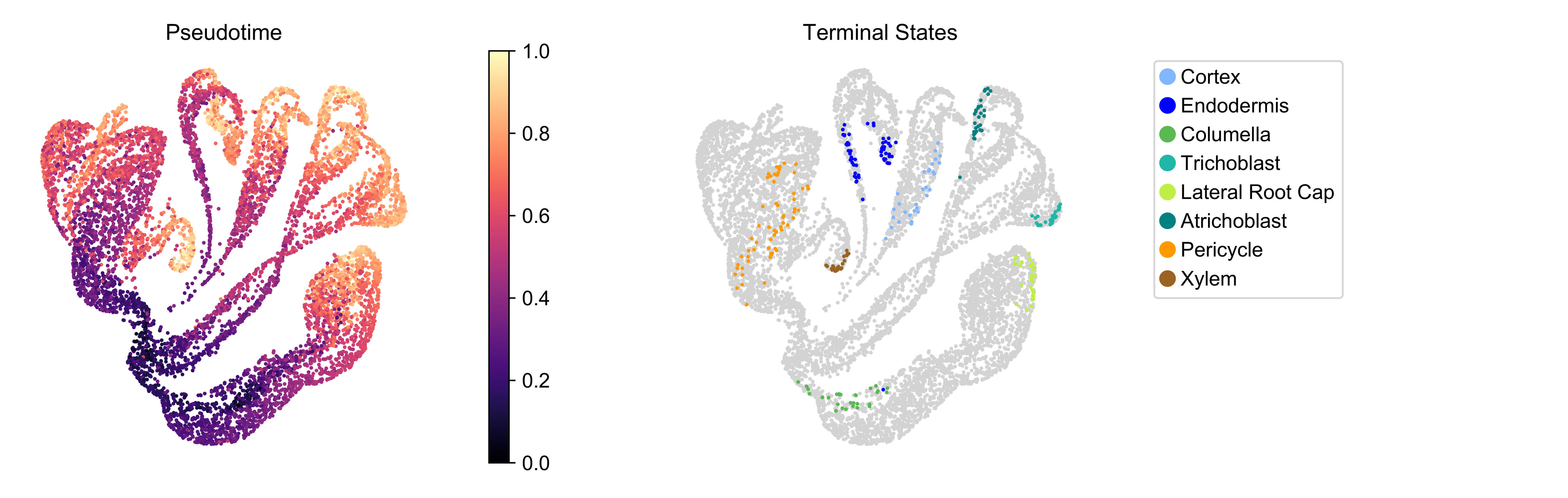

Supplement: S4 Fig — Terminal states found using automatic detection functionality offered by the CellRank package, coloured by their corresponding lineage (right). No terminal states were identified for the phloem and procambium lineages. Additionally, as is clear from pseudotime (left), some states that are intermediate are miss-classified as terminal. (TIF) [file pcbi.1009466.s006.tif]

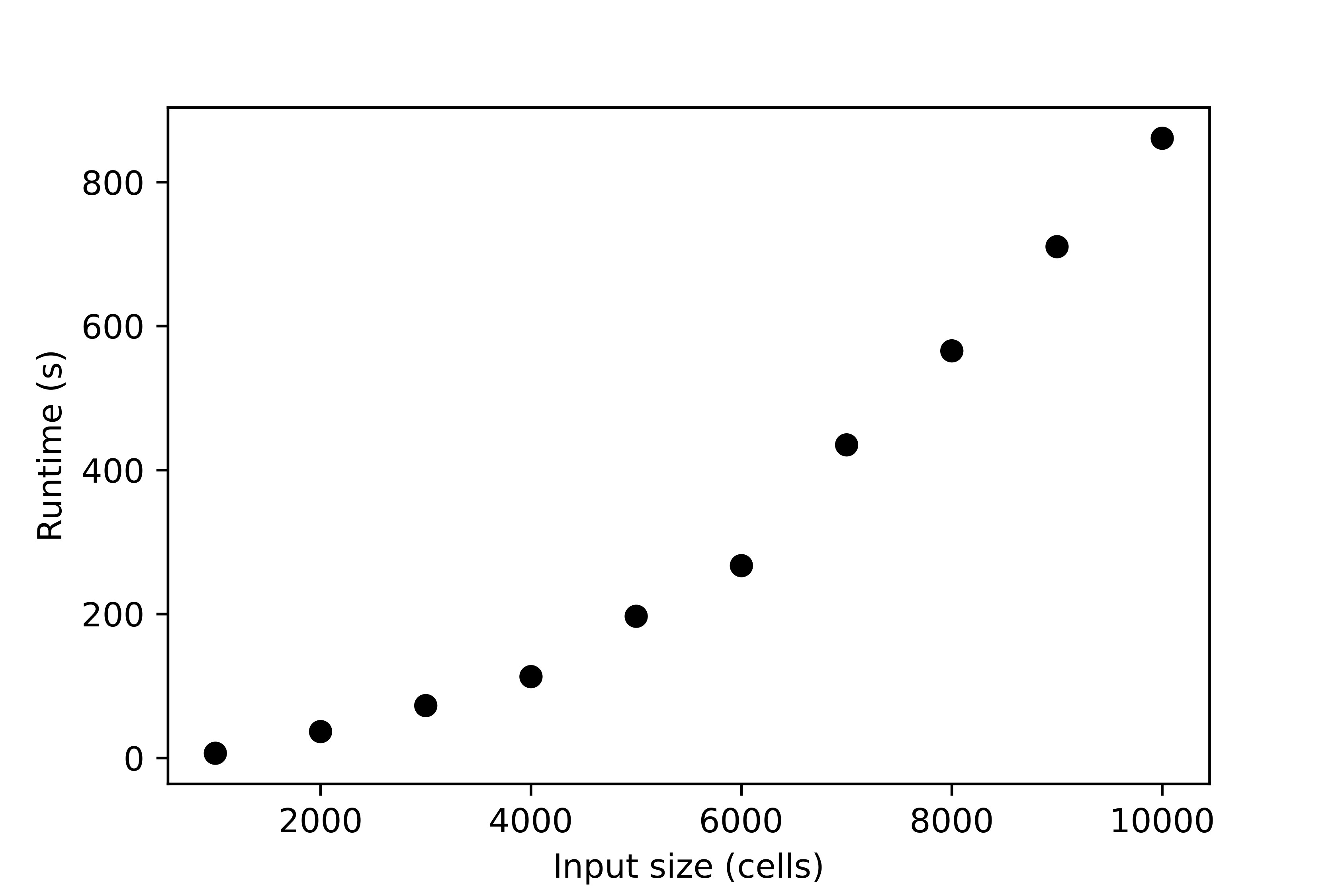

Supplement: S5 Fig — (TIF) [file pcbi.1009466.s007.tif]
