# Supplementary material for: Optimal transport analysis reveals trajectories in steady-state systems
Source: PLoS Comput Biol. 2021 Dec 3;17(12):e1009466. doi: 10.1371/journal.pcbi.1009466 (PMC8691649; doi:10.1371/journal.pcbi.1009466)
Supplement: Attachment — Submitted filename: ZAGMS_submission_v3_reviewer_comments.pdf [file pcbi.1009466.s009.pdf]

We are grateful to the referees for lending their time and expertise in reviewing our manuscript. Their input has been greatly appreciated and we have made significant revisions to our manuscript to incorporate the feedback. We begin below with an overview of the main issues raised by the reviewers and then provide point-by-point replies to each reviewer.

Reviewer 1

We thank Reviewer 1 for their positive comments about the manuscript, including:
... they provide a quite distinct perspective/approach, and this development will be highly interesting to the community of optimal transport, and also the community of single-cell method development.

The reviewer's key requests concern two main points:
- **Theoretical justifications of the splitting scheme approach from which the method is derived.** We have now revised the text to make the justification for this choice clearer.
- **Clarification of the potential landscape assumption.** We have added an illustrative example (see Supplement A.1) in the revised text that clarifies why this assumption is necessary.

Reviewer 2

We thank Reviewer 2 for their positive comments about the manuscript, including:
Overall, I found this to be a well founded and valuable contribution to the trajectory inference literature from scRNA-seq data.

The reviewer's key requests concern two main points:
- **Discussion of the growth/flux rate estimates and their impact on the performance of the method.** We have now performed additional numerical experiments with simulated and real data to investigate the sensitivity of the method to errors in the input rates, and we have revised the text to discuss our results.
- **Discussion of the method in the context of related literature.** Following the reviewer's suggestions, we have rewritten Section 1.4 "Related work" to address the existing literature more comprehensively.

Reviewer 3

We thank Reviewer 3 for their positive comments about the manuscript, including:
While there is a great abundance of trajectory inference tools, the authors have motivated the novelty and added value of their method well. Its ability to incorporate RNA velocity is also a huge plus.

The reviewer's key requests concern two main points:
- **Discussion of how the method relates to/could interoperate with dynamo.** We have now cited the Dynamo paper and mentioned how our method could relate to it. We note in particular that the downstream dynamical system/differential geometry analysis offered by dynamo could be applied to vector fields recovered by StationaryOT.
- **More reader-friendly introduction to the mathematical content of the paper.** To make the

introduction more accessible to readers, we have added a new figure (Figure 1 in revised text) that portrays the mathematical concepts relevant to our method.

Detailed Point-by-Point Response

Reviewer 1

Inferring differentiation dynamics at the single cell level from single-cell measurements is an important and active area of research. Among many existing approaches, optimal transport has been previously shown, by one of the authors, to provide good estimates for differentiation dynamics from single-cell measurements profiled at different time points. They now demonstrate that optimal transport can also be when there is only a snapshot of the single cell measurements, provided that the system is stationary and a rough estimate of the growth and death rate is available at different regimes of the manifold. They show that this inference is well-justified theoretically when the differentiation dynamics is driven by a potential landscape, while also providing a heuristic extension to non-equilibrium setting. In the latter case, the inference is only qualitatively correct. The authors benchmark their method with existing methods, like PBA, and show comparable performance. However, they provide a quite distinct perspective/approach, and this development will be highly interesting to the community of optimal transport, and also the community of single-cell method development. Overall, this paper is very well written. I therefore highly recommend the publication of this paper after the authors fix the following issues.

Response: We thank the reviewer for their time and detailed comments on our manuscript.

Major comments:
I think it is quite a clever idea to split the dynamic equation (2) into the growth equation and also the drift-equation. This makes the application of OT becomes natural. Intuitively, I think it makes a lot of sense, but I just wonder if there is a simple proof that the split is equivalent to the original equation? I think adding this proof will make the paper more solid theoretically.

Request: The reviewer asks whether there is a simple proof of the validity of the splitting scheme in equations (4-6).
Response: Indeed there is: we have revised the text to discuss the convergence and consistency of the splitting method of equations (4-6). We summarise the theoretical arguments below.

We agree with the reviewer that the theoretical justification of this splitting approximation is important.

The solution to the PDE Eq. (2), is approximated by the solution of the system Eqs. (4-6) up to second order pointwise, i.e. $O(\Delta t^2)$. This is a well known result for Lie splitting in the literature of numerical partial differential equations (see e.g. [15, Section 1.3]). Thus, in the limit as Δt shrinks to zero, the splitting approximation converges to the true solution.

The point of using the splitting approximation is that we separate transport (i.e. the spatial differential operators for diffusion and drift) and growth (i.e. the effects arising from the source term). Thus if the growth is characterised, then we can use optimal transport to explain the remaining transport dynamics.

Drift and diffusion scale as $O(\Delta t)$ and $O(\sqrt{\Delta t})$ respectively, and so asymptotically (i.e. for small enough Δt), the $O(\Delta t^2)$ error introduced by the splitting approximation is negligible.

Although it makes sense that the inference would work exactly only for a dynamic system driven by a potential field, I could not see this limitation directly. If you start from a population equation that is equivalent to (2), but also allows rotation (non-potential term), you can still perform the splitting as in Fig. 1. And the argument that it should be diffusion-driven at small interval is still valid. Then, you can still apply optimal transport as defined in (8). Maybe I missed it. Could you clarify this in the main text?

Request: The reviewer asks for us to clarify the need for the potential landscape assumption discussed in Section 1.3.
Response: We thank the reviewer for this suggestion, and we added a section (Supplement A.1) providing a detailed discussion for why this assumption is necessary.

If we do not assume that the system is potential driven, then in general there are multiple vector fields that can give rise to the same stationary population distribution. We agree that a self-contained explanation will be helpful, although this same assumption has been discussed in related works such as [47]. We have revised the text to include an illustrative example in Supplement A.1, referred to on page 3.

For people to use the package, it would be useful if you could provide a plot showing the computational cost in a standard laptop as a function of the cell number.

Request: The reviewer asks for an additional plot showing computational cost vs. number of cells.
Response: We thank the reviewer for this suggestion, and we have generated the requested figure.

See Supplementary Figure 26, in which we have recorded the compute time for the Arabidopsis example on a standard CPU-only Google Colaboratory instance (Xeon 2.30Ghz x2, 12 GB RAM). We believe this is illustrative of a standard end-user use case.

Related to Fig 10, please provide a plot systematically showing the effect of lambda on the inference.

Request: The reviewer asks for an additional plot for fate correlation vs. lambda in Figure 10.
Response: We thank the reviewer for this suggestion, and we have generated the requested figure.

We have added the requested figure as Figure 10(c) in the revised manuscript and have also added a discussion of the results on page 13 (~lines 342-345).

Also, did you try StationaryOT to the hematopoiesis data that PBA was originally applied? This is just a suggestion. You can decide whether to do this or not, although I think this application will make the package more appealing, and more people would want to try it out.

Response: We thank the reviewer for suggesting this. It is certainly a good idea, and we intend to add this as a tutorial example in the GitHub repo in the future.

We explored applying StationaryOT to the original hematopoiesis dataset from the PBA paper. However, the annotation of cells by source / sink that was provided by the PBA paper was insufficient for our purposes. Compared to PBA, our method requires a larger number of sink cells (~100 vs ~10). We see this as a strength of our method, because the results are robust to the choice of source / sink cells.

While the PBA paper does provide the gene signature used to identify terminal cells, the publicly available expression matrix does not include identifiers for individual genes. We plan to reach out to the authors to try to obtain a version of the data that includes gene names and will then add a tutorial with the dataset to the Github repo.

Minor comments:
In many places the color bar are missing. Please fix that.

Response: We have added a color scale in Figures 4, and 10.

We will note that in Figures 3(a) and 9(a), we omit the color scale and now show instead the contours of the potential. This is purely for illustrative value, since we want to focus on the geometry of the potential rather than the actual values (which can be very large, making it difficult to show on a color scale).

*Similarly, the x-label, and y-label are often too difficult to understand. It would be useful to provide a verbal explanation before the annotation. For example, in Fig. 8a, I do not understand what is X*U.*

Response: We thank the reviewer for pointing this out -- Indeed the explanation of x.u was neglected. We have now updated the figure caption.

Since the recovered process lives in the 10-dimensional space X and we want to visualise it in a 2-dimensional plot of state vs. time, we show only a single-dimensional projection along a subspace u that is chosen to show the three branches.

In the section ‘’Laws on paths”, I do not understand how the trajectory is generated for each single cell (Fig. 8), and also what is the W2 distance for? Is it a distance between path probability distribution from different methods (i.e., inferred by Stationary-OT and ground-truth)? I think part of the problem is that many notations are introduced without explanation. For example, what is f, g and T in the equation above Fig. 8. Please fix this.

Response: We thank the reviewer for commenting on this. We have addressed the points raised in the main text, and have also added information on the definition of the W_2 distance in Supplement A.3.

- We now discuss the sampling of trajectories in lines 289-292. In summary, we start with an initial particle state $X_0 = x_0$ at t = 0 and construct a trajectory by repeatedly sampling from the time- Δt transition density.
- Yes, the W_2 distance is between path probability distributions (see Supplement A.3).

Reviewer 2

The authors present a new method for computing trajectories in a steady state systems with a single population snapshot using optimal transport. This method uses the fact that at the steady state equilibrium the population drift plus the growth rate is in equilibrium, i.e. equal at all points. They use this to infer the population drift, represented as a Markov process over the cells, from prior knowledge on the growth rates or source and sink states of cells. This is effectively the inverse of the problem tackled in velocyto and scVelo, which infer sources and sinks from velocity / transition information. Overall, I found this to be a well founded and valuable contribution to the trajectory inference literature from scRNA-seq data.

Response: We thank the reviewer for their time and detailed comments on our manuscript.

Major comments:

The authors claim: "We show that optimal transport analysis, a technique originally designed for analysing time-courses, may also be applied to infer cellular trajectories from a single snapshot of a population in equilibrium. "

However, a key component of their method requires prior estimates of the growth rate / sink probability of each cell. In fact all of the experiments are performed in the case where we have access to accurate estimates of growth rates, which is not a trivial piece of information to extract from scRNA-seq data. For the root tip scRNA-seq experiment the authors use growth rates estimated from imaging, which is a relatively reliable piece of side information that is not (in general) present in most scRNA-seq experiments. To be more widely applicable it would be important to demonstrate that this method works with unreliable estimates of the growth rate, preferably attainable from Transcriptome data only. Therefore, either the main claim should be modified to inferring trajectories from a snapshot with prior knowledge of per cell growth rates, or additional experiments should be performed to validate the method when growth estimates are more unreliable and realistic.

Request: The reviewer asks for further discussion/investigation of the role that growth estimates play in the performance of the method.
Response: We thank the reviewer for making this point and agree that this is an important question. We have updated the manuscript accordingly with discussion and results for both simulated and Arabidopsis datasets.

In the setting of simulations, in Section 2.6 of the initial submission we investigated mis-specification of the flux proportions in Figure 7. This examines the sensitivity of results to the estimates of the flux rate at the sinks, and we originally argued that there was generally a mild dependence. We have since revised Figure 7(b) to now show more extensive results, where the ground truth proportions were subject to the full range of possible perturbations. We discuss this in the accompanying text, noting that for a reasonably wide range of perturbations the correlation remains high.

For the Arabidopsis dataset, we agree that the use of imaging data in our case is application specific. We have now added an extended discussion of growth rates in Supplement B.2. Using only transcriptomic data, cell cycle signatures could be used to estimate growth rates. This approach was used successfully by Schiebinger et al. [32]. We applied a modification of this cell cycle estimation scheme to the Arabidopsis data, and compared to the results obtained using imaging-derived growth rates. We found that using cell-cycle growth rates resulted in results comparable to those we present in the main text.

For the sensitivity comparison of entropic vs. quadratic regularized OT it would be good to mention the relative size of H(\gamma) and | \gamma |_2^2, as if they are of significantly different magnitude (on these examples) then this would have a confounding effect on the sensitivity to epsilon. Otherwise this is quite an interesting observation.

Request: The reviewer asks for us to clarify how values of \epsilon are compared between entropic and quadratic regularised OT.
Response: We thank the reviewer for this suggestion, and we have updated the text to clarify this point.

We are glad the reviewer found this aspect of our work interesting. We would like to draw attention to Figure 7(a) in which ε is displayed on a logarithmic scale. We made this choice in order to remove any differences in scale of ε between the two choices of regularised OT. From this, we hope it is clear that quadratic OT is less sensitive to changing ε by a log-fold change. We have updated the text to make this clearer.

A few notes on related work, it would be useful to draw distinctions between the methods benchmarked in [1] and the trajectory terminology used here. As there are so many different methods for inferring trajectories, it is important to understand where this particular method is most applicable. [2,3] also learn the potential landscape based on approximating the SDE, but do so from time series in continuous (but relatively low dimensional) space. Here the authors approximate the SDE over the empirical distribution. It would be helpful to include some discussion on the costs / benefits of this discrete approximation. Furthermore, [2] discusses estimation of the drift from a single observation of the steady state equation and the drawbacks of this type of inference. It would be useful to add this type of discussion in light of more recent trajectory inference methods. In light of these related works, to my knowledge this method is novel, particularly in building a Markov process from a single snapshot based on growth rates over the support of the empirical distribution.

1. Saelens, W., Cannoodt, R., Todorov, H. & Saeys, Y. A comparison of single-cell trajectory inference methods. Nature Biotechnology 37, 547–554 (2019).

2. Hashimoto, T. B., Gifford, D. K. & Jaakkola, T. S. Learning Population-Level Diffusions with Generative Recurrent Networks. in Proceedings of the 33rd International Conference on Machine Learning 2417–2426 (2016).

3. Sisan, D. R., Halter, M., Hubbard, J. B. & Plant, A. L. Predicting rates of cell state change caused by stochastic fluctuations using a data-driven landscape model. Proceedings of the National Academy of Sciences 109, 19262–19267 (2012).

Request: The reviewer asks for us to add further discussion of our method in the context of related work.
Response: We thank the reviewer for suggesting improvements in this area. We have revised Section 1.4 [Related work] to provide a broader discussion of related work and address those comments raised by the reviewer.

We address the three works mentioned by the reviewer below:

- The methods benchmarked by Saelens et al. are devoted to the problem of recovering the topology of the dynamical process. This can be thought of as a coarse-grained approach to trajectory inference. Our method deals with the problem of inferring dynamics at the single-cell level, and so falls into a different class of methods. See lines 105-110 in revised text.
- The approach of Sisan et al. deals in low dimensions and involves a density fitting step for estimating the continuous population density (see [Sisan et al. 2012, Materials and methods]), and is thus related to the classical approach of estimating the potential as the negative logarithm of steady-state density. See lines 99-104 in revised text.
- Hashimoto et al. develop a method for learning continuous vector fields from time-series datasets, which is quite different from the scenario we consider. In particular, the vector field is parameterised by a neural network and thus only finding a local minimum is guaranteed. As we mention in Section 1.3, optimal transport allows us to solve a convex problem for the transition matrix, i.e. we are guaranteed to find a unique solution.

Minor comments

1.2: "Typically we will take X = R^d", is this true? From my understanding this paper takes X as the support of the data, \bar{X}

Response: This is the ambient state space for the abstract model which we start from (it must be a continuous space for the SDE to make sense). However, we have updated the text to use \subset.

On line 146 you switch between \mu_0, \mu_1 notation and \mu, \nu notation.

Response: Thank you for pointing this out, this has been corrected.

There is an error in the equation above line 205, z_1 is repeated twice, and the z_2 should probably be cos(3 \pi / 2) as depicted in Figure 3.

Response: Thank you for pointing this out, this has been corrected.

What are the units of the estimated MFPT in Figure 6(a), Markov process steps? Or is it normalized by \delta t?t's repeated.

Response: Thank you for pointing this out, these are in Markov chain steps. We have updated the corresponding figure caption to reflect this.

Line 471 mentions 15-20 minutes for computation on 10,000 cells, it would be nice to mention the hardware used here as this computation is quite hardware sensitive. Thank you for mentioning the hardware used for the full atlas.

Response: The 15-20 minutes figure was for the full atlas, but we notice that our choice of wording might be confusing. We have rearranged the order of sentences to clarify this (see line ~516). For subsampled datasets, we have added a plot of the compute time as a function of number of cells (see Figure 26), along with a description of the hardware used.

Given that you identified sparsity as a key benefit of quadratic regularization, did you try the unregularized OT formulation? This would obviously not scale particularly well, but may be of interest on small examples if sparsity in the transportation matrix is beneficial.

Response: This is an excellent question: unregularised OT would not be able to capture the stochastic nature of the process. We explain in detail below.

Our motivation for using entropy-regularized optimal transport is the connection to Brownian motion and SDEs (see the discussion around line 181-183). Ordinary (unregularized) OT would correspond to the zero noise limit of the SDE, and the corresponding transport plans would be essentially deterministic. Since biological processes involve some level of intrinsic noise, this is not necessarily desirable.

We find empirically that quadratic OT produces transport plans that introduces sparsity in a less stringent way which still allows for some stochasticity in the transport plan, see e.g. [23]. We do not know if the precise connection to SDEs also applies to quadratic OT, this would be an interesting avenue for future work.

Reviewer 3

In this paper Zhang et al. introduce the use of optimal transport (OT) for trajectory inference in tissues where the differentiating cells are in equilibrium, noting its success in inferring trajectories of cells in synchronous development systems such as embryogenesis. Theynoting its success in inferring trajectories of cells in synchronous development systems such as embryogenesis. They frame the problem in the context of differential equations at the cell- and population level. The latter measures the change of population densities of cells at a given point in gene expression space as a function of time. An equilibrium state implies setting this population balance PDE equal to zero, which is achieved by sources and sinks in the vector field, that can be interpreted as stem cells dividing to generate new developing cells (sources), and cells either dying or leaving the tissue after maturation (sinks). Optimal transport is used to find a coupling between two cells after a small time step, accounting for these sources and sinks.

While there is a great abundance of trajectory inference tools, the authors have motivated the novelty and added value of their method well. Its ability to incorporate RNA velocity is also a huge plus.

This paper would be a valuable addition to PLOS Computational Biology, but some comments must be addressed first.

Response: **We thank the reviewer for their time and detailed comments on our manuscript.**

Major comments:

I see many similarities with dynamo (https://github.com/aristoteleo/dynamo-release) but it is not referenced in the manuscript. This warrants a comparison, at least in the introduction. Also, can they be integrated by using the vector field recovered by dynamo, which can exploits RNA metabolic labeling and protein acceleration?

Request: The reviewer asks (i) for a comparison to Dynamo, at least in the introduction and (ii) if the information from Dynamo can be integrated.
Response: We thank the reviewer for directing our attention to Dynamo, which we now cite and discuss in the revised introduction (see lines 55-61).

(i) (Comparison) Dynamo estimates a vector field of single cells using RNA velocity and other techniques (e.g. metabolic labelling), as opposed to methods such as StationaryOT and PBA which do not necessarily require RNA velocity as an input. One consequence of this is that methods such as StationaryOT and PBA can be applied to data from CyTOF and ATAC-seq experiments, where it is not straightforward to estimate velocity in the usual way.
(ii) (Integration) The Dynamo vector field could be used as a prior velocity field to StationaryOT (as could also be done with methods such as CellRank, scVelo). Also, the various downstream analysis methods offered by Dynamo could be applied to the vector field output by StationaryOT.

A welcome addition to the paper would be an additional section in the introduction to explain the relevant concepts at a higher level. The jump from section 1.1 to 1.2 is currently too large for many readers to appreciate the methodology. Terms such as “potential landscape” and “vector field” should be explained here, as right now they are suddenly mentioned in section 1.1 (line 54) without any explanation. The new section should ideally also explain how OT (section 2.2) is related to the differential equations (1.2). Example papers that use OT in single cell technology with digestible explanations of the methods are novoSpaRc (Nitzan et al., 2019) and SpaOTsc (Cang and Nie, 2020). See also the first results section / Box 1 in the dynamo preprint (Qiu et al., 2021) for a high-level explanation on what is a vector field and what can be done with it.

Request: The reviewer asks for us to provide additional discussion of the relevant concepts for this paper, in order to make the introduction more reader friendly.

Response: We thank the reviewer for this suggestion and we agree that parts of the introduction could be made more reader friendly. We have revised the preliminary sections of the text to incorporate this feedback (see below).

(i) In order to provide readers with a gentler introduction to the mathematical content we consider, we have added a concept figure (Figure 1) that provides a visual summary of the inference problem. It

illustrates the concept of vector field, trajectories, population snapshots, transition probabilities and fate probabilities, which are the key mathematical objects we discuss.

(ii) The connection between OT and differential equations dates back to the work of Schrodinger [33]. The goal of our discussion in this paper is to motivate the application of OT. For the interested reader looking for rigorous statements of the theory, we have added a pointer to several references (see lines ~181-183 in revised text).

Minor comments:

Figure 1 needs more explanation in the caption. Please walk the reader through the figure as the idea of matching the two marginal distributions is what motivates the use of OT.

Response: We thank the reviewer for pointing this out. We have updated the figure caption to explain the splitting scheme in more detail. Note that the relevant figure is now Figure 2.

Solution p_G above figure 1: what is g(x)? I don't see it defined and its not referenced anymore.

Response: We thank the reviewer for pointing this out. We defined implicitly g(x) = exp(R(x)), and have now updated the text to state this explicitly.

L134: "the dynamics are Markov". Is my understanding correct that this implies that the cell state at timepoint t+delta t is fully determined by the cell state at time t? I.e. we do not need previous states (markov property). Is it therefore assumed that a cells next state can be predicted fully from its current state, or is this well known? Please elaborate this Markov part and if my understanding is correct, state the assumption more clearly or source the claim. With RNA velocity we can approximate X_t+delta t. We can use this to capture the transitions of the cells. As you explain in the paper, the main issue is that we do not have the cells state at time t+delta t. So to what extent does this simplify the methodology?

Request: The reviewer asks us to elaborate on the need for a Markov assumption, and its implications for analysis.

Response: We thank the reviewer for the questions. We provide a detailed response below, and we have revised relevant parts of the text.

By "Markov dynamics" we mean that the transition densities depend only on current cell state. The Markov assumption corresponds to using the SDE/Fokker-Planck formulation of the stochastic process (1). In many ways, a Markov assumption is a necessary one in order to make any kind of efficient analysis possible (e.g. simulation, computing fate probabilities, etc.). Essentially the same assumption is used (at least implicitly) by methods such as CellRank, PBA, scVelo, etc.

Since we make the Markov assumption, knowing the transition probability over some time dt allows us to predict the system's state at future times {dt, 2*dt, 3*dt, …} by composing the transition matrices. That is,

the system's behaviour is characterised by the transition matrix alone (and we learn this transition matrix using optimal transport).

We have updated the section "1.2 Modelling assumptions" to state and justify the Markov assumption more clearly.

Related to this, it is not immediately obvious how RNA velocity can be used, nor is it actually applied in the Arabidopsis thaliana root tip experiment as far as I can tell. Please clarify.

Response: For the Arabidopsis application, applying RNA velocity (using scVelo+CellRank) resulted in a substantial region of vectors that pointed in the opposite direction to the known biological progression. This gave us reason to expect that incorporating a prior RNA velocity field could worsen the inference instead of improve it. Since in simulation we demonstrated that good velocity data can improve results, we believe that RNA velocity could be useful for our method in other datasets.

Line 48: "To date, the methods developed for these two different paradigms have remained largely distinct, with time-course methods generally sharing little in common with methods designed for stationary systems. In this paper we show that optimal transport analysis, a technique originally designed for analyzing time-courses [29], may also be applied to infer cellular trajectories from a single snapshot of a population in equilibrium. Therefore, optimal transport (OT) provides a unified approach to inferring trajectories, applicable to both stationary and non-stationary systems."
Clarify what makes a method uniquely applicable for each problem type. The ability to add prior information? I am familiar with GrandPrix (Ahmed et al., 2019) and Ouija (Campbell and Yau, 2019) where a prior can be set on the pseudotime to be roughly the same as capture time.

Request: Related to our claim that most trajectory inference methods fall into one of two categories (analyzing time-courses vs the stationary setting), the reviewer asks us to clarify what makes a method uniquely applicable to each problem type.

Response: We agree -- the statement we made was too broad given the diversity of methods developed to date. Thus, we have removed this assertion in the revised text (see line ~48 in revised text).

To answer the reviewer's specific question for clarification, our remark pertained specifically to "topology-free" inference methods, where cellular dynamics are represented by a cell-cell transition kernel (e.g. PBA, CellRank, scVelo, Waddington-OT, Dynamo). This is in contrast to "topology" methods which seek to capture the topology of paths along the valley floors of Waddington's landscape (e.g. PAGA, SlingShot, Wishbone, GrandPrix, etc.).

I appreciate how the growth rates and sink locations are well-motivated in the Arabidopsis thaliana root tip example by referring to the imaging study. However I suspect in many cases this information might not be available. Can you supply fallback mechanisms for such cases? For example, the sinks might be identified using the diffusion end points as done in the original RNA velocity paper (La Manno et al., 2018, fig 3c).

Response: If only transcriptomic data is available, growth rates can be estimated by cell cycle score. We have added an additional analysis (Supplement B.2) where we estimated the growth rates for Arabidopsis thaliana using a variation of the cell cycle score method applied in the original Waddington OT paper (Schiebinger et al., 2019).

While we expect all cells in the meristem to divide, only some would be dividing during a single snapshot. We used the cell cycle signature to estimate the proportion of cells dividing for each lineage and then applied this growth rate to all cells in the meristem. Despite differences in the imaging and cell cycle growth rates, we found 79% and 78% of cells to match the annotation using the cell cycle growth rates and entropic and quadratic regularization respectively, at most a 3% degradation from the imaging growth rates which achieved 80% and 81% matches.

In the root tip example, all non-sink cells are set to be sources, which if my understanding is correct implies that these cells are actively dividing and therefore introduce new particles into the system. From the original paper I understand that only the cells within the meristem divide: "Young, dividing meristematic cells are at the base of each branch followed by elongating and finally mature, differentiated cells at the tips(Fig 1c)". Please clarify, or, if this decision was a practical one, users would appreciate this as a practical tip in the github repository.

Response: Thank you for pointing this out. Indeed there was an inaccuracy in how we referred to 'sources' and 'sinks'. We have modified the text (see line ~398) to correct this.

In the Arabidopsis data, cells which were not assigned to be sinks were given a growth rate *g*. Non-meristem cells were assigned *g = 1* which corresponds to no division or death, as you have correctly pointed out.
