# Supplementary material for: Optimal transport analysis reveals trajectories in steady-state systems
Source: PLoS Comput Biol. 2021 Dec 3;17(12):e1009466. doi: 10.1371/journal.pcbi.1009466 (PMC8691649; doi:10.1371/journal.pcbi.1009466)
Supplement: Attachment — Submitted filename: review.docx [file pcbi.1009466.s008.docx]

In this paper Zhang et al. introduce the use of optimal transport (OT) for trajectory inference in tissues where the differentiating cells are in equilibrium, noting its success in inferring trajectories of cells in synchronous development systems such as embryogenesis. They frame the problem in the context of differential equations at the cell- and population level. The latter measures the change of population densities of cells at a given point in gene expression space as a function of time. An equilibrium state implies setting this population balance PDE equal to zero, which is achieved by sources and sinks in the vector field, that can be interpreted as stem cells dividing to generate new developing cells (sources), and cells either dying or leaving the tissue after maturation (sinks). Optimal transport is used to find a coupling between two cells after a small time step, accounting for these sources and sinks.

While there is a great abundance of trajectory inference tools, the authors have motivated the novelty and added value of their method well. Its ability to incorporate RNA velocity is also a huge plus.

This paper would be a valuable addition to PLOS Computational Biology, but some comments must be addressed first:

Major

I see many similarities with dynamo (<https://github.com/aristoteleo/dynamo-release>) but it is not referenced in the manuscript. This warrants a comparison, at least in the introduction. Also, can they be integrated by using the vector field recovered by dynamo, which can exploits RNA metabolic labeling and protein acceleration?

A welcome addition to the paper would be an additional section in the introduction to explain the relevant concepts at a higher level. The jump from section 1.1 to 1.2 is currently too large for many readers to appreciate the methodology. Terms such as “potential landscape” and “vector field” should be explained here, as right now they are suddenly mentioned in section 1.1 (line 54) without any explanation. The new section should ideally also explain how OT (section 2.2) is related to the differential equations (1.2). Example papers that use OT in single cell technology with digestible explanations of the methods are novoSpaRc (Nitzan et al., 2019) and SpaOTsc (Cang and Nie, 2020). See also the first results section / Box 1 in the dynamo preprint (Qiu et al., 2021) for a high-level explanation on what is a vector field and what can be done with it.

Minor

Figure 1 needs more explanation in the caption. Please walk the reader through the figure as the idea of matching the two marginal distributions is what motivates the use of OT.

Solution p_G above figure 1: what is g(x)? I don't see it defined and its not referenced anymore.

L134: "the dynamics are Markov". Is my understanding correct that this implies that the cell state at timepoint t+delta t is fully determined by the cell state at time t? I.e. we do not need previous states (markov property). Is it therefore assumed that a cells next state can be predicted fully from its current state, or is this well known? Please elaborate this Markov part and if my understanding is correct, state the assumption more clearly or source the claim.

With RNA velocity we can approximate X_t+delta t. We can use this to capture the transitions of the cells. As you explain in the paper, the main issue is that we do not have the cells state at time t+delta t. So to what extent does this simplify the methodology? Related to this, it is not immediately obvious how RNA velocity can be used, nor is it actually applied in the *Arabidopsis thaliana* root tip experiment as far as I can tell. Please clarify.

Line 48: "To date, the methods developed for these two different paradigms have remained largely distinct, with time-course methods generally sharing little in common with methods designed for stationary systems. In this paper we show that optimal transport analysis, a technique originally designed for analyzing time-courses [29], may also be applied to infer cellular trajectories from a single snapshot of a population in equilibrium. Therefore, optimal transport (OT) provides a unified approach to inferring trajectories, applicable to both stationary and non-stationary systems."

Clarify what makes a method uniquely applicable for each problem type. The ability to add prior information? I am familiar with GrandPrix (Ahmed et al., 2019) and Ouija (Campbell and Yau, 2019) where a prior can be set on the pseudotime to be roughly the same as capture time.

I appreciate how the growth rates and sink locations are well-motivated in the *Arabidopsis thaliana* root tip example by referring to the imaging study. However I suspect in many cases this information might not be available. Can you supply fallback mechanisms for such cases? For example, the sinks might be identified using the diffusion end points as done in the original RNA velocity paper (La Manno et al., 2018, fig 3c).

In the root tip example, all non-sink cells are set to be sources, which if my understanding is correct implies that these cells are actively dividing and therefore introduce new particles into the system. From the original paper I understand that only the cells within the meristem divide: “Young, dividing meristematic cells are at the base of each branch followed by elongating and finally mature, differentiated cells at the tips(Fig 1c)”. Please clarify, or, if this decision was a practical one, users would appreciate this as a practical tip in the github repository.
